# Modelling lipid systems in fluid with Lattice Boltzmann Molecular Dynamics simulations and hydrodynamics

**DOI:** 10.1038/s41598-019-52760-y

**Published:** 2019-11-11

**Authors:** Astrid F. Brandner, Stepan Timr, Simone Melchionna, Philippe Derreumaux, Marc Baaden, Fabio Sterpone

**Affiliations:** 1 CNRS, Université de Paris, UPR 9080, Laboratoire de Biochimie Théorique, 13 rue Pierre et Marie Curie, F-75005 Paris, France; 2 Institut de Biologie Physico-Chimique-Fondation Edmond de Rothschild, PSL Research University, Paris, France; 3 ISC-CNR, Dipartimento di Fisica, Università Sapienza, P.le A. Moro 5, 00185 Rome, Italy; 4 Lexma Technology 1337 Massachusetts Avenue, Arlington, MA 02476 USA

**Keywords:** Computational biophysics, Computational models

## Abstract

In this work we present the coupling between Dry Martini, an efficient implicit solvent coarse-grained model for lipids, and the Lattice Boltzmann Molecular Dynamics (LBMD) simulation technique in order to include naturally hydrodynamic interactions in implicit solvent simulations of lipid systems. After validating the implementation of the model, we explored several systems where the action of a perturbing fluid plays an important role. Namely, we investigated the role of an external shear flow on the dynamics of a vesicle, the dynamics of substrate release under shear, and inquired the dynamics of proteins and substrates confined inside the core of a vesicle. Our methodology enables future exploration of a large variety of biological entities and processes involving lipid systems at the mesoscopic scale where hydrodynamics plays an essential role, e.g. by modulating the migration of proteins in the proximity of membranes, the dynamics of vesicle-based drug delivery systems, or, more generally, the behaviour of proteins in cellular compartments.

## Introduction

The study of biomembranes based on experimental, theoretical and computational approaches occupies a central place in modern biophysics. These structures constituted by lipids of different types as well as other molecules (e.g., proteins, sterols and carbohydrates), allow the structural organisation of living matter by creating the outer envelope of cells and their intracellular compartments^1^. A biomembrane is a highly heterogeneous *milieu* where the different types of lipids and membrane proteins, which can account for nearly 50% of the total mass, are partitioned non-uniformly, giving rise to domains of varying structural order and internal dynamics^2^. Being more than mere protective walls, biomembranes form a highly dynamic environment hosting numerous essential metabolic and signalling processes. Furthermore, lipid bilayers that mimic cellular membranes are used by bioengineers as a basis for novel drug-delivery constructs and artificial organelles^3,4^. Only recently have we started to grasp the full complexity of these lipid environments, giving rise to the field of lipidomics^5^.

Nowadays, a great variety of experimental techniques allows characterising many aspects of the structure and dynamics of biomembranes, e.g. the extension of membrane domains^6^, lipid diffusion^7^, protein localisation^8^, membrane deformation and fusion^9^. Moreover, membrane constructs such as vesicles are studied experimentally for their transport and content release capabilities^10,11^. Their behaviour under flow has been studied to assess deformations^12^ and mechanical-force-induced stress effects^13^.

A strong support to understanding membrane systems is provided by computer modelling and simulation. During the last 30 years, the development of atomistic force fields combined with the availability of massive computer power have boosted the investigation of systems of growing size and complexity^14^. This evolution has made it possible to capture processes ranging from membrane permeation of small solutes^15^, pore formation^16^ up to the dynamics of a whole respiratory complex^17^. While the popularity of atomistic simulations of membrane systems is benefiting from improvements in algorithms and code efficiency, including the use of enhanced sampling techniques^18,19^, the length- and time scales associated with many membrane-related phenomena require the use of coarse-grained (CG) models^20–24^. A large body of biophysical processes has been investigated via CG models as reported in many in-depth review articles^14,25^. Some examples include the study of mobility of lipids and proteins in membranes^26–28^, membrane protein aggregation^29^ and clustering^30^, and vesicle fusion^31^.

The success of CG models in exploring very complex processes relies on the substantial reduction in the number of degrees of freedom to handle computationally. Moreover the smoothness of the CG energy landscape enables the use of significantly increased integration timesteps. In this regard, implicit solvent models^31–34^ are particularly attractive, but, by default, they cannot describe the effect of dynamical correlations mediated by the solvent. However, these correlations not only play an important role in the aggregation process of lipids^35^, and other biomolecules^36^, but may critically influence biomolecule migration in the proximity of membranes^37^ as much as vesicle mobility and encounters^38^. In the “Modelling Manifesto” put forward by Lyman, Hsieh and Eggeling^39^ this aspect was carefully discussed.

Here we present a coupling of Dry Martini^31^, an implicit-solvent coarse-grained lipid model, with the Lattice Boltzmann Molecular Dynamics (LBMD) technique^40–43^ to include hydrodynamic interactions (HI) in the implicit-solvent simulation of membranes. This work represents an alternative to a recently published coupling of Dry Martini to the multi-particle collision method^44^ and extends our previous work where the OPEP implicit solvent model was coupled to HI^36,43,45–48^. We show here that this coupling represents a general tool to treat the impact of HI in a broad range of processes involving lipids, not only standard bilayer membranes, but also, for instance, to investigate the dynamics of a vesicle under the perturbative action of the external fluid flow. This technique will allow synergic interactions with experiments where lipidic systems are manipulated in fluid flows^49,50^. In fact, the microscopic character of Dry Martini, the efficiency and the natural multi-scale character of LB to treat a fluid makes the LBMD an appealing method. LB can also handle fluids of different nature such as charged species in water in order to solve electrokinetic problems^51^. This feature, already tested for instance to characterise DNA translocation in pores^52^, can be extended to investigate voltage-dependent processes at the proximity of neuronal membrane systems.

## Results

### The implementation of Dry Martini

**Figure 1:**
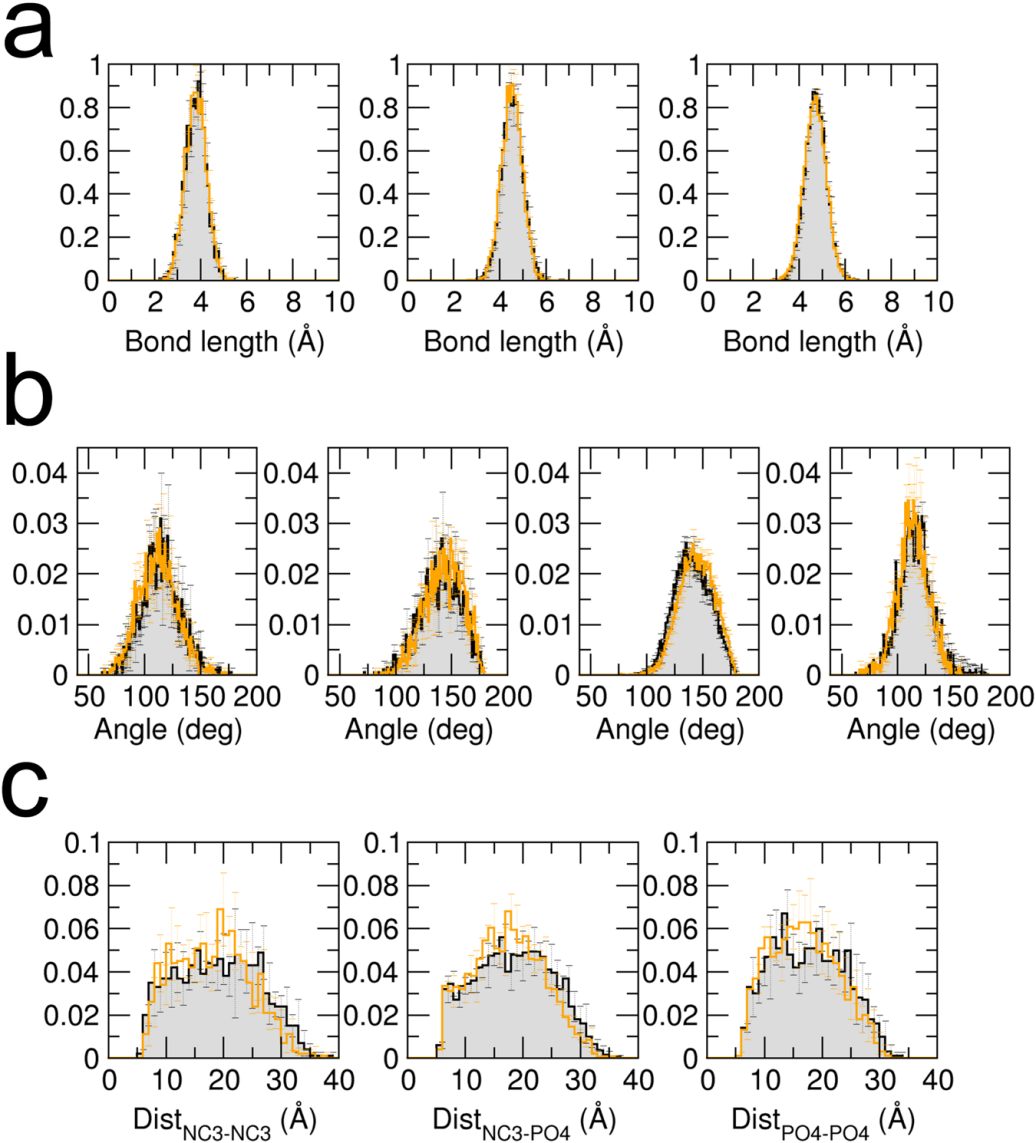
Probability distributions of bonds, angles and charged beads distances. Black lines and gray surfaces represent results obtained from a simulation carried out using Gromacs, and the orange lines represent the results from our implementation in MUPHY. **(a,b)** Probability distributions of each of the unique bond or angle types present in Dry Martini respectively. **(c)** Probability distributions of the distances between charged beads (NC3: choline bead, PO4: phosphate bead).

In this section we present a test of the numerical implementation of the force field in the software MUPHY^53^. We verify that the structural properties governed by the intra- and intermolecular interactions reproduce those obtained from the original implementation in the Gromacs software (version 4.5.5). A dimer of POPC lipids was simulated by standard MD simulation for a few nanoseconds with temperature controlled by the Langevin thermostat and set to $310\text{K}$. The probability distributions of the values taken by representative degrees of freedom in the dimer are plotted in Fig. 1 and compared to those extracted from a reference simulation carried out using Gromacs. The results show excellent agreement.

### Coupling with HI

**Figure 2:**
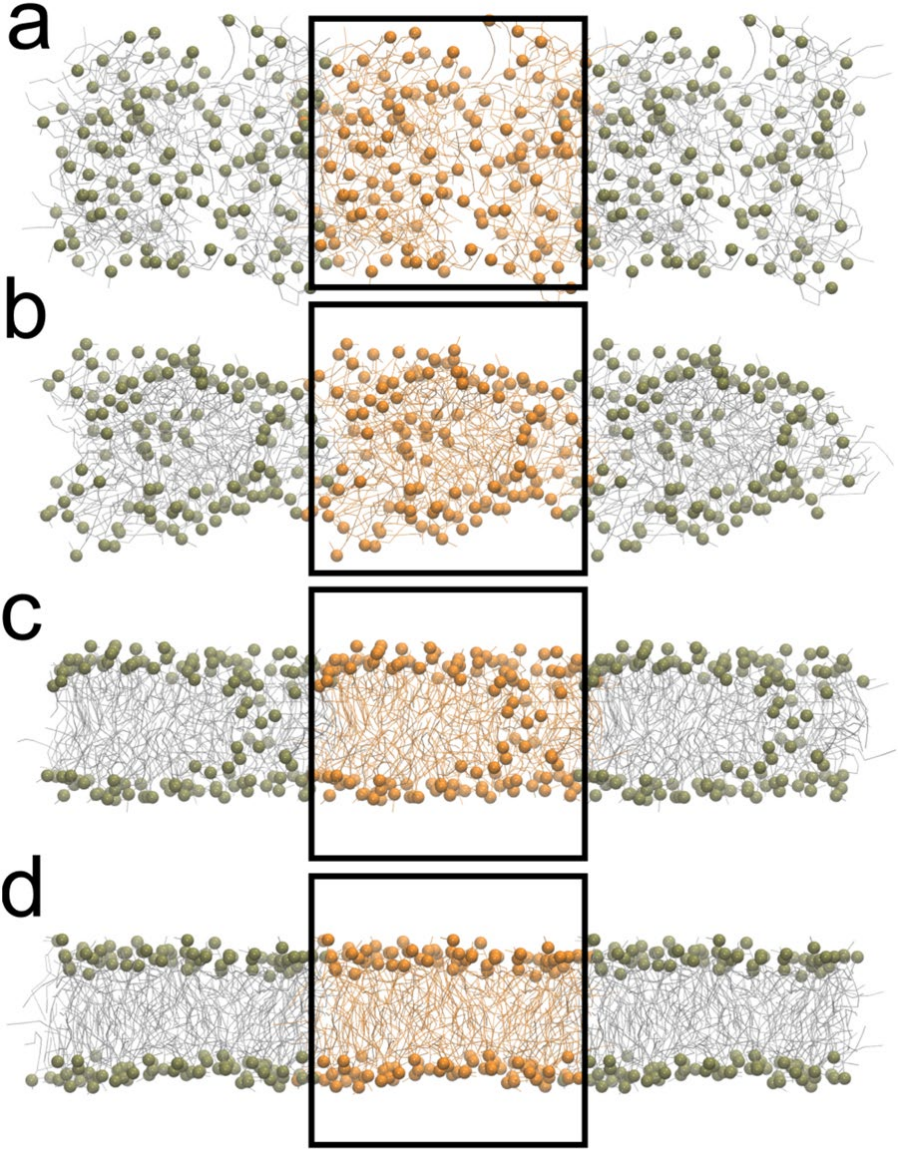
Spontaneous bilayer assembly of 128 POPC lipids. The (panels a–d) show the time evolution of the assembly process. For the sake of clarity, the phosphate beads are represented as van der Waals spheres, whereas the rest of the lipid is shown as lines. The system is represented with periodic images, and the orange-coloured particles belong to the simulation box.

The second type of tests was designed to inquire the effect of the hydrodynamic coupling on the global properties of a simple lipidic system. We have first verified that the coupling with LBMD does not alter the structural properties of a formed bilayer. We performed simulations with and without HI of a membrane bilayer oriented perpendicular to the Z-axis with dimension in the X-Y plane ranging from $10\times 10$ nm^2^, up to $40\times 40$ nm^2^. For all systems, the measured average thickness, $\simeq 38$ Å , is unaffected by the coupling with the external fluid. It is also worth noting that the fast fluctuations of the membrane thickness occurring on the picosecond timescale are not affected by HI, see SI Fig. S1. We have also computed the radial distribution function for the hydrophobic beads of the lipids inside the membrane and verified that the inclusion of HI does not alter the local packing either, see SI Fig. S1. This is in agreement with the work presented in ref.^44^ where it is shown that the inclusion of HI via the STRD technique has a negligible impact on the thermodynamic properties of a lipid membrane modelled using Dry Martini. Finally, a small system composed by 128 POPC lipids randomly placed in a cubic box of size $L=75$ Å was simulated with and without HI to explore the role of HI on the self-assembling of the lipids. In both cases a bilayer was formed, see Fig. 2 for a pictorial representation.

The timescale of the assembly process clearly depends on the choice of the friction parameter $\gamma$. For $\gamma =2.5\times 1{0}^{-4}$ fs^−1 ^a complete bilayer structure was obtained in about $10$ ns, while for higher values of $\gamma$ the kinetics was slower. When formed, the final thickness of the bilayer was between 38–39 Å which is in agreement with experimental data^54^. Interestingly, for this highly concentrated small system HI have negligible impact on the aggregation process, which is rather controlled by the mechanical forces. To be noted, however, that in the investigation of other molecular systems (amyloid peptides, or simple lipids) at lower concentration it was demonstrated that HI accelerate the aggregation process^35,36^.

### Lipid lateral diffusion

**Figure 3:**
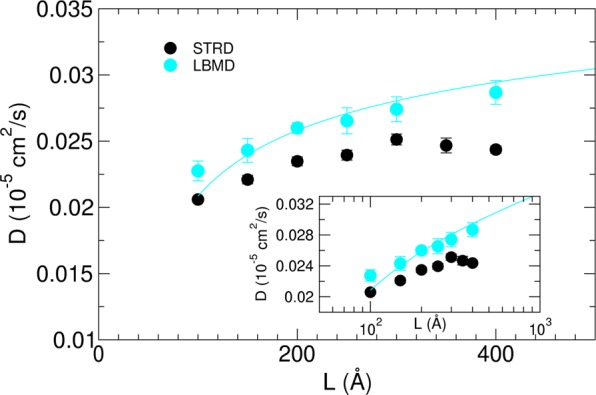
Box size effect on lipid diffusion. Diffusion coefficient for POPC lipid extracted from 1.2–1.5 μs simulation of a planar membrane in different boxes of size ${L}_{x}={L}_{y}=L$ and ${L}_{z}=240$ Å (cyan circles). For each system the last part of the trajectory (800 ns) was divided into independent blocks of 100 ns. The diffusion coefficient D was obtained for each block from a linear fit of the MSD using a time window of 20-40 ns for the fit. The averages and standard errors were computed over the independent blocks. The continuous line represents a fit of the data performed according to the theory proposed in ref.^55^. The fit was done using the bulk fluid viscosity set in LBMD, and the characteristic size of a lipid molecule $R=0.5$ nm. We also assumed the value of the interleaflet friction to be equal to what was obtained for the same CG model by Lyman *et al*.^44^ ($b=2.87\cdot 1{0}^{5}$ P cm^−1^). The membrane-surface viscosity we derived from the fit is ${\eta }_{m}=5.09\cdot 1{0}^{-8}$ P cm. In the figure we also report the data from ref.^44^ based on STRD simulations of the POPC lipid planar membrane with inter-membrane separation of $200$ Å (black circles). Inset: Log-linear plot of the diffusion coefficients.

Here we focus on lipid dynamics inside a membrane. Recently, the effect of periodic boundary conditions (PBC) and of simulation box size on the calculation of the lipid lateral diffusion in simulations has been thoroughly investigated, and rationalised^27,55,56^. Because of reentrant hydrodynamic interactions under PBC (stemming from both the solvent and lipids), it was shown that the calculated diffusion constant for lipids (or an embedded rigid body like a protein) in a planar membrane simulated with periodic boundary conditions ($${D}_{PBC}$$) diverges logarithmically as a function of the lateral box size $${L}_{x}={L}_{y}=L$$^27,55,56^ for fixed transversal separation ($${L}_{z}$$). It was however shown that the value of the diffusion coefficient for an infinite system ($${D}_{0}$$) to be compared with experiments can in principle be obtained using adequate numerical procedures^27,55,57^. In previous studies the size dependence on the calculated lipid diffusion was estimated by considering either explicit solvent atomistic models or the explicit solvent CG model Martini^27,55,56^. Recently, a work by Lyman *et al*.^44^ reported the coupling of the implicit CG Dry Martini model with the multiple-collision algorithm, also referred to as Stochastic Thermostatted Rotation Dynamics (STRD)^58^, in order to integrate solvent mediated hydrodynamic interactions in implicit solvent particle simulations. With this coupling in hands the authors studied the size dependent lipid lateral diffusion in POPC membranes. The lateral dimensions of the membrane system were increased from $${L}_{x}={L}_{y}=L=100$$ Å up to $$400$$ Å while the separation between two periodic membranes in the transversal direction was $$200$$ Å. In the same spirit we performed LBMD simulations of POPC membranes of different sizes ($$100$$ Å $$ < L < 400$$ Å) as in ref.^44^. We set $${L}_{z}=240$$ Å to ensure a separation of $$ \sim 200$$ Å between two periodic membranes along the transversal direction as in ref.^44^. For each system the simulation was 1.2–1.5 μs long, the last 800 ns of the trajectories were used for calculating the 2D mean square displacement (MSD) via block analysis. In Fig. 3 we report the calculated average diffusion coefficients from the LBMD simulations and compare to the results from STRD^44^. Our data reproduce the size dependency already observed for Dry Martini and using STRD^44^, with the diffusion constant growing unbounded with the lateral box size, and start showing the logarithmic divergent behaviour as predicted by theory (see the inset graph of the Fig. 3 where we show the log-linear plot of the diffusion coefficient D). Our finding shows that LBMD is a computationally appealing alternative strategy for using implicit solvent CG lipid models coupled to the hydrodynamic interactions from an embedding fluid. The approach allows to simulate large sized membrane systems at a cheaper computational cost with respect to explicit solvent CG models. A complete investigation of the effect of sizes (membrane dimension as well as the transversal separation) and fluid viscosity, as for instance presented in refs^27,55,56^, is beyond the scope of the present modelling and it is reserved for a future study.

### Nanotube adsorption

The contribution of HI stemming from the surrounding fluid can be appreciated by considering the adsorption dynamics of a biomolecule on the membrane surface. Two aspects should be considered, the first concerns the general effect of HI on biomolecule diffusivity^35,36,43,59^. For a single protein, and even for aggregated objects, it was previously shown that the inclusion of HI speeds-up molecular transport. This effect can be rationalised^35^ for instance by referring to the Zimm description of polymer dynamics^60^ where with the inclusion of HI, the diffusivity scales more favourably with the polymer size than the correspondent case without HI (Rouse description^61^). The second aspect, which we do not explicitly explore here, relates to the bending oscillations of the lipid membrane that couple with the solvent dynamics and cause a repulsive effective force on particles moving along the membrane surface^37^.

**Figure 4:**
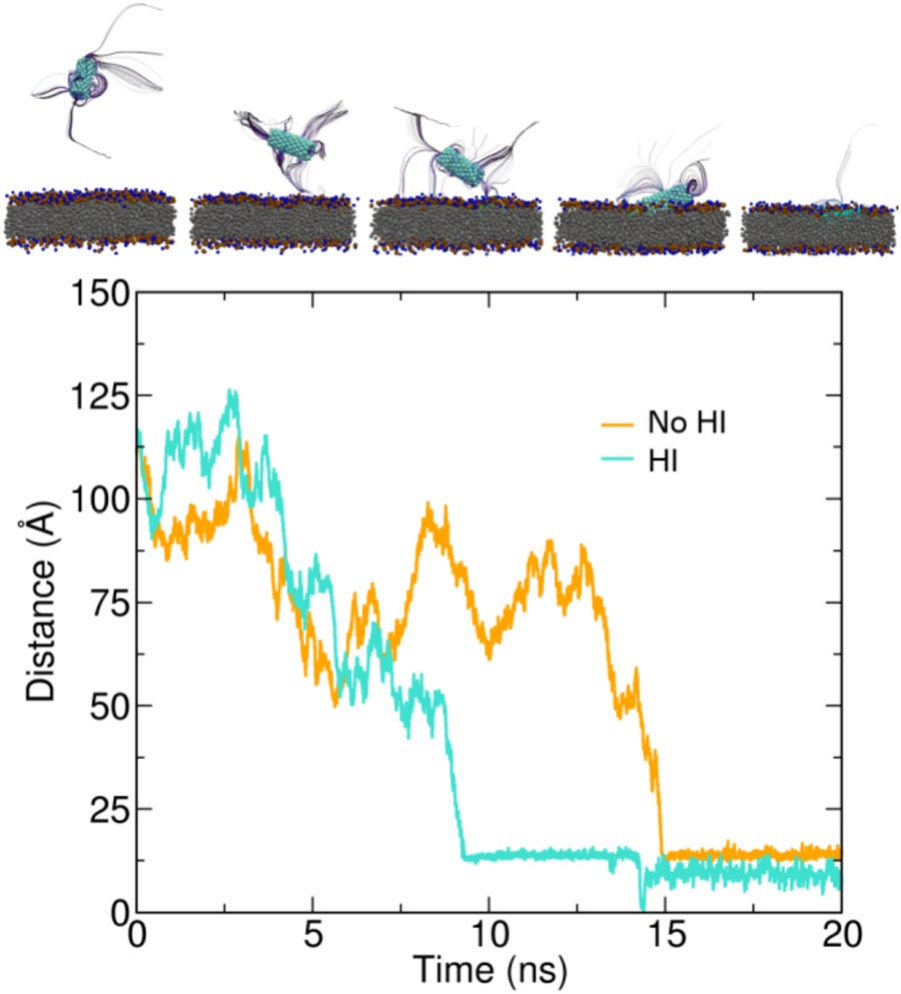
Nanotube adsorption. Time evolution of the minimal distance of the nanotube centre of mass with respect to the external surface of the lipid bilayer for one of the sets of initial conditions. Starting from the same initial condition two simulations were performed with (cyan) and without (orange) HI. In the top part of the figure we represent the streamline of the fluid surrounding the nanotube during its adsorption process.

Here, as a simple test, we considered the adsorption process of a nanotube of length $L=43.1$ Å and radius $r=7.5$ Å on the surface of a flat membrane in a simulation box of lateral sizes ${L}_{x}={L}_{y}=150$ Å and transversal dimension ${L}_{z}=300$ Å. Several independent orientations of the nanotube were generated by rotating its axis and maintaining the centre of mass at a distance of $113$ Å from the membrane surface. In our simulations the dynamics of adsorption is generally accelerated by the presence of HI interactions, with the average adsorption time being $10 \%$ shorter than without hydrodynamics, see SI Table S1. Since at the considered length scales the membrane does not exhibit visible transversal oscillations, we do not observe hydrodynamic repulsive effects stemming from the membrane motion^37^. An example of the adsorption process is represented in Fig. 4 where the time series of the minimal distance between the centre of mass of the nanotube and the membrane surface is plotted for a simulation including or neglecting hydrodynamics. A pictorial representation of the nanotube dynamics including the surrounding fluid represented by its isokinetic streamlines is presented in the top part of the figure.

### Vesicles in fluid flows

The capability to represent effectively a lipid membrane together with the surrounding solvent fluid opens up the possibility to investigate systems and processes of biological and technological relevance. One example is the behaviour of vesicles and liposomes under shear flow^62–67^. Vesicles are simple models of cellular compartments, and their response to a shear flow has been studied to understand both the response of large biological structures, such as red blood cells^62^, or the design of drug delivery vectors^63–66^.

Recently, it was shown experimentally that a vesicle, depending on external fluid viscosity and shear rate, can experience three different dynamical regimes, from tank treading to tumbling and trembling^12,68^. The first two motions imply a preserved shape while the third one is associated to shape deformations. To show that the LBMD method can reproduce the behaviour of vesicles under a shear flow we have assembled a small POPC spherical vesicle formed by $394$ lipids ($R \sim 34$ Å) immersed in a fluid in a cubic simulation box of size $L=240$ Å. We observed how an external shear flow affects its dynamics and shape fluctuations. We scanned four different values of the shear rates, $\dot{\gamma }=3,7,20,30\times 1{0}^{9}$ s^−1^, which allowed us to monitor the response of the systems on the nanosecond time scale. In refs^47,69^ the interested reader can find the technical details about how a shear flow is generated. To be noted that shear rates used here compare to the values applied in recent DPD simulations to inquire the effect of shear on protein migration in the membrane of vesicles^67^.

**Figure 5:**
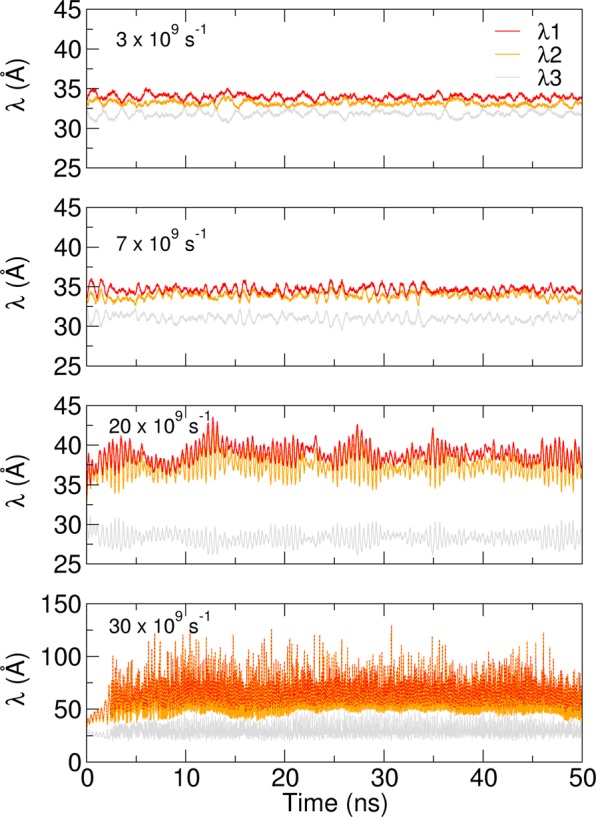
Vesicle in shear flow. Time evolution of the three eigenvalues of the vesicle gyration tensor for shear rates $\dot{\gamma }=3,7,20,30\times 1{0}^{9}$ s^−1^.

In the simulations, the increase of shear rate causes a progressive deformation of the spherical vesicle towards an prolate shape. This transformation is quantified by the eigenvalues ${\lambda }_{i}$ of the vesicle gyration tensor (${\lambda }_{1}\ge {\lambda }_{2}\ge {\lambda }_{3}$), see Fig. 5, and their relative ratio (see SI Fig. S3). The shear rate not only causes a deformation but also controls the oscillation of the gyration eigenvalues ${\lambda }_{i}$. This frequency of the oscillation of ${\lambda }_{i}$ increases due to the rotational component of the shear field, with characteristic time inversely proportional to the shear rate, $\tau \propto 1/\dot{\gamma }$. When the shear field becomes too high the vesicle breaks apart, and the lipids start forming dynamical entities which encounter, partially fuse and dissociate as they follow the fluid flow. A pictorial representation is reported in SI Fig. S5.

**Figure 6:**
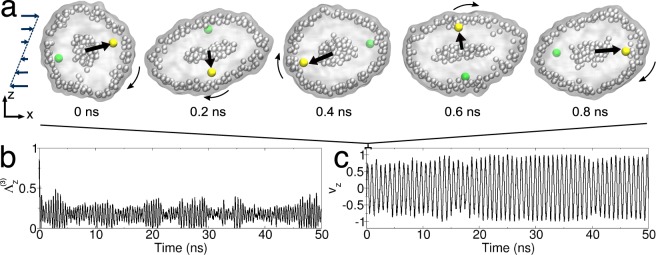
Vesicle in shear flow. In the top layer of the figure **(a)** we represent the short time rotational motion of the vesicle under shear flow. Two lipid beads corresponding to the outer layer are highlighted (yellow and green spheres) to help visualising the membrane rotation. The yellow and green beads remain aligned at a short time, but at a longer time, as we show in SI Fig. S4, the different lipid mobility in the bilayer causes their misalignment. In panels **(b)** and **(c)** we represent the time evolution of the normalised projection on the Z-axis of the system of two characteristic vectors of the vesicle: the eigenvector ${\Lambda }^{(3)}$ of the gyration tensor and associated to the longest axis of the prolate **(b)**, and the vector ${\bf{v}}$ connecting one polar bead of the lipids in the membrane (yellow sphere) to the centre of mass of the vesicle **(c)**. For the projection of the eigenvector we considered the absolute value.

In the Fig. 6a we have represented the typical evolution of the vesicle, and its membrane, during a period of the sliding dynamics ($$\sim 1$$ ns). In order to characterise the dynamics of the lipids and the vesicle, we have projected the eigenvector $${\Lambda }^{(3)}$$ of the gyration tensor along the Z-axis along which the velocity gradient is aligned in the Couette flow. The eigenvector $${\Lambda }^{(3)}$$ is associated to the main axis of the prolate. For this projection we considered the absolute value. Similarly we have considered the vector $${\bf{v}}$$ connecting one polar bead of a lipid in the membrane to the centre of mass of the vesicle. For this vector too, we have considered the projection along the Z-axis. The normalised projections are reported in Fig. 6b,c and show two characteristic motions. The first motion concerns the oscillation of the main axis of the vesicle. After a transient initial phase, when the vesicle is deformed by the flow, the main eigenvector $${\Lambda }^{(3)}$$ of the gyration tensor never aligns with the Z-axis, in fact the value of the projection stays always $$ < 0.5$$. The second motion relates to the sliding of the membrane which has a slower frequency with respect to the oscillation dynamics of the vesicle principal axis.

**Figure 7:**
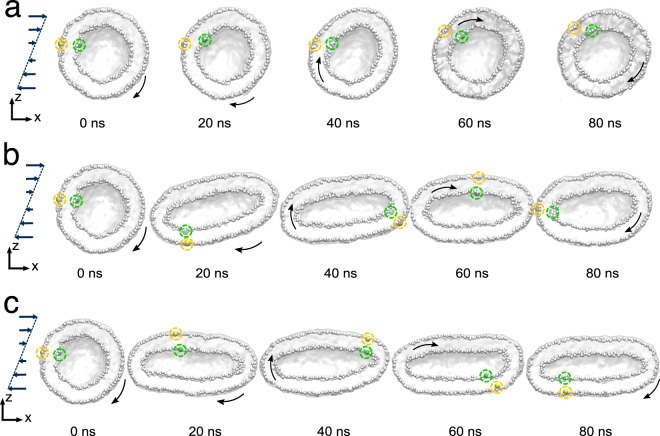
Vesicle in shear flow. Time evolution of the shape of a larger vesicle ($R \sim 100$ Å) under the action of the shear flow. **(a–c)** Simulations at different shear rate: $\dot{\gamma }=3.7\times 1{0}^{7}$ s^−1^, $1.9$ and $3.7\times 1{0}^{9}$ s^−1^, respectively. Two lipid beads corresponding to the outer and inner layer are highlighted (yellow and green spheres, respectively) to help visualising the membrane rotation and the lipid motion inside the membrane.

In order to model a more a realistic system and match the characteristic size of POPC small unilamellar vesicles^70^ we have performed extra simulations of a larger vesicle of radius $\simeq 100$ Å, and formed by 3451 lipids. The vesicle was placed in a cubic simulation box of size $L=450$ Å under the action of a shear flow of rate $\dot{\gamma }=1.9$ and $3.7\times 1{0}^{9}$ s^−1^ and $\dot{\gamma }=3.7\times 1{0}^{7}$ s^−1^. Again, as seen in the previous example, in Fig. 7 we observe the shape deformation toward a prolate structure for high shear values (Panel b and c), while for this vesicle a shear rate $\dot{\gamma }=3.7\times 1{0}^{7}$ s^−1^ does not affect the spherical shape. The dynamics of the vesicle’s characteristic vectors is reported in SI, Fig. S6.

The response of a vesicle to the external shear flow^63,64^ can be exploited to design drug delivery systems or biological constructs that control chemical reactivity. We therefore considered an extra system -a small toy model- to test how the release of substrates can be simulated using our mixed particle/fluid approach. In this model a spherical POPC vesicle formed by $n=923$ lipids with an initial radius $R=60$ Å was filled with a small number of substrates, $15$ small alanyl-phenylalanyl-alanine tripeptides. The substrate was modelled as an elastic network, see the Methods section, and the interaction parameters with the membrane were inspired by characteristic energy terms of the OPEP force field. The vesicle was placed in a cubic box of size $L=350$ Å under a shear flow with $\dot{\gamma }=6\times 1{0}^{9}$ s^−1^.

**Figure 8:**
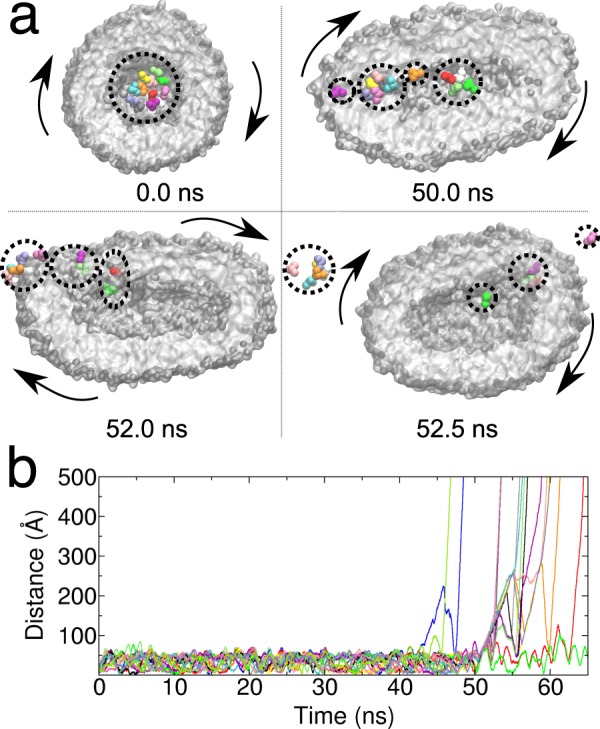
Substrate release in shear flow. Time evolution of the single substrate distance with respect to the vesicle centre of mass. In the top part of the figure we represent the molecular detail of the substrate release. Clusters of substrate molecules are highlighted by dashed lines. Arrows indicate the tumbling motion of the vesicle.

As discussed above, a spherical vesicle undergoes deformation by the shearing of the surrounding fluid, and acquires prolate conformations. As a consequence, the internal pool is deformed as well and the substrates under the action of the external perturbation accommodate in the flattened space, see Fig. 8. After 40 ns, some of the substrates penetrate the membrane and are released in the solution. At the very beginning the evacuation involves just a few molecules but under the constant stress of the external fluid, a collective release eventually occurs. It is interesting to note that the substrate release does not require a disruption of the vesicle but it is the consequence of a forced permeability of the membrane. It seems that the drag caused by the external shear flow results in an effective force that induces the substrate to cross the membrane. In a separate work, we will inquire the molecular basis of the evacuation process by considering larger and realistic systems, and by investigating the different aspects controlling the process, e.g. how the vesicle shape deformation alters the local packing of the lipids in the membrane and changes the associated free-energy barrier for membrane penetration.

### Nanoreactor

In order to demonstrate that the LBMD technique and the presented coupling with the Dry Martini lipid force field can be used to study very large systems, we considered a more complex construct aimed at modelling a vesicular nanoreactor. In fact, vesicles filled with enzymes and substrates are widely used to investigate biochemical reactions under confinement^71^. Here we employ the LBMD approach to simulate the first detailed model of a vesicular nanoreactor, inspired by constructs investigated in previous experimental studies^72,73^. We selected $\alpha$-chymotrypsin, one of the enzymes reported to exhibit increased activity under confinement, as the enzyme to be placed inside the nanoreactor. The nanoreactor was formed by a POPC vesicle with an inner radius of $120$ Å, placed in a cubic simulation box of size $L=410$ Å. The vesicle is filled with 5 $\alpha$-chymotrypsin molecules (238 residues) in the presence of 6 protein crowders (BSA, 583 residues), corresponding to a crowder concentration of 92 g/L, accompanied by 100 oligopeptide molecules (alanyl-phenylalanyl-alanine), forming the substrate for the enzymatic reaction.

**Figure 9:**
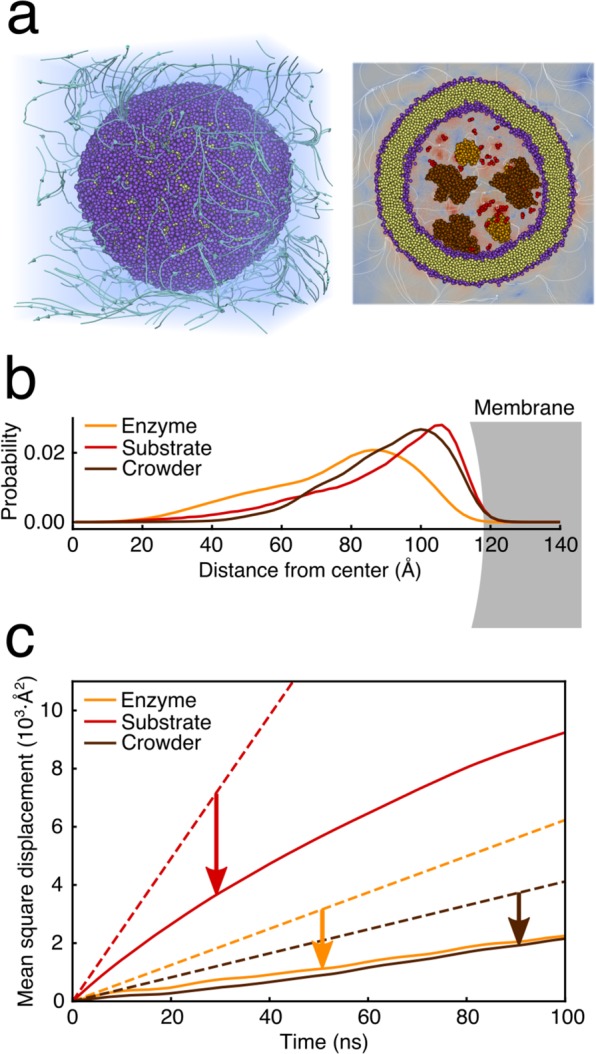
Nanoreactor. **(a)** Pictorial representation of the nanoreactor and the internal and external fluid. **(b)** Probability distribution of proteins and substrate localisation inside the vesicle. **(c)** Proteins and substrate mean square displacement in dilute condition (dashed lines) and inside the vesicle (solid lines).

Preliminary simulation (300 ns) allowed us to characterise the spatial distributions of the various molecules inside the nanoreactor as well as their diffusivities (see Fig. 9). These factors affect the rate of the chemical reaction by governing the probability that an enzyme will meet a substrate. We observed that both the protein crowders as well as the substrate molecules were concentrated near the lipid membrane (Fig. 9b), with the large protein crowders diffusing tangentially to the membrane surface. The enzymes also interact with the lipid bilayer. However, rather than being in direct contact with the membrane surface, some of the enzymes preferred to bind to the crowder, which brought them closer to the centre of the vesicle. For all the three molecular species, we observed a slowdown in diffusion compared to dilute conditions in the absence of confinement. On the short timescale (1–5 ns), the slowdown is 35–50 depending on the molecule type. On the longer timescale (10–50 ns), the slowdown of the diffusion became even more pronounced ($\sim 60 \%$), reflecting the finite size of the vesicle interior and the interactions with the lipid membrane. For the BSA proteins, the slowdown inside the vesicle was larger by $30 \%$ than that measured via neutron scattering for a BSA solution at comparable crowding condition^74^. Interestingly, at the long timescale, the diffusion coefficient of the enzymes practically matched that of the significantly larger crowders (see Fig. 9c), as a consequence of their mutual interactions in the vicinity of the membrane. Finally, we did not observe any major changes in the shape of the vesicle in the course of the simulation, with the maximum deviations not exceeding 4–6 % of the vesicle size in each dimension. The results of our simulation confirm the capacity of the LBMD technique to provide a detailed molecular view of protein motion in large and realistic systems consisting of many biomolecules surrounded by a lipid membrane. Thus, the results pave the way for simulations of entire organelles or therapeutic constructs to capture the complex biological processes occurring in their interior.

## Conclusions

In this work we coupled the coarse-grained Dry Martini model for lipids^31^ to the Lattice Boltzmann Molecular Dynamics technique^41–43^. This is a precious combination since it naturally allows including hydrodynamic interactions while still preserving the computational gain related to the implicit solvent nature of the Dry Martini model. It was already demonstrated that the presence of solvent mediated correlations is a necessary ingredient to study biological systems at the mesoscale^35,36,75,76^, including important processes involving membranes, such as lipid aggregation^35^, protein migration in proximity of membranes^37^, vesicle encounter and fusion^38^.

We first validated the implementation of the force field in the multi-scale simulation software MUPHY^53^. Then, we explored different situations where the coupling with the surrounding solvent is key for the behaviour of the lipid system. For instance, we examined the size effect in the estimate of lipid diffusion. In these regards, the LBMD technique based on Dry Martini could be computationally appealing with respect to explicit solvent models in order to approach the infinite-size limit necessary to calculate the transport properties of lipids or proteins in two-dimensional membrane systems^27,55,56^. Since the technique allows simulating a great variety of fluid flow conditions, we used the methodology to explore the behaviour of a vesicle under the external perturbation of shear flow mimicking experimental assays^12,63,64^. Finally, inspired by recent experimental work^71^ we explored the possibility to simulate a nanoreactor so as to investigate the combined effect of confinement, crowding and hydrodynamics on substrate/enzyme encounters.

The presented coupling could be further extended. All our simulations were performed in the canonical ensemble at fixed volume and temperature. However, for a membrane system it is well known that it is important to control the lateral pressure to ensure the accurate structural and dynamical behaviour specifically for laterally periodic systems^77,78^. In our scheme it is possible to implement an efficient algorithm for the particle dynamics to sample the isobaric ensemble. The fluctuating simulation cell would be mapped on a fixed number of grid points used for the resolution of the fluid dynamics. According to our tests, at equilibrium even for a small membrane system (${L}_{x}={L}_{y}=50$ Å), where the fluctuations are expected to be most pronounced, they remain below 2% of the mean lateral size. This implies that cell fluctuations modify the grid spacing by about 2% as well. When preparing a system in isobaric condition the transient relaxation leads to a shrinking of the cell that is on the order of 4% of the initial lateral size. Therefore, even in this case, one can utilise a constant resolution for the LB component.

A second aspect to mention concerns the application of multi-resolution meshes to increase efficiency. Our scheme is suitable for the study of transport properties in two-dimensional membrane systems in periodic boundary conditions where in order to approach the infinite-size limit the transversal dimension should be increased enormously. The computational cost increases linearly with the size of the transversal dimension with a single grid spacing, but a tremendous gain can be achieved by using a multi-resolution approach, where only a portion of the space surrounding the membrane is resolved at the fine scale while the remainder of the volume is mapped on a coarser grid. Also, the computation of the particle dynamics can be boosted by implementing new algorithms to exploit the vectorial capability of modern CPUs^79^ and approaches for parallelism to handle more efficiently the sparse nature of some lipidic systems like large vesicles.

In conclusion, in this work we extended the operational capability of our multi-scale Lattice Boltzmann Molecular Dynamics technique^36,43,46–48^, an alternative to the scheme proposed by Lyman where Dry Martini was coupled to the STRD method^44^. With our approach we are now able to explore a great variety of biophysical processes involving lipid systems at the mesoscale where solvent mediated interactions are important. Namely, our approach can support the bio-engineering and biotechnology experimental investigation of lipidic systems like vesicles in fluid flow conditions^49,50^. In a forthcoming work we will concentrate on the effective coupling between the Dry Martini force field and the coarse-grained model for protein OPEP in order to model complex protein/lipid systems with a flexible model for proteins.

## Methods

In this section we detail the implementation of the Dry Martini force field and its coupling with the Lattice Boltzmann Molecular Dynamics technique. An overview of the setup of the systems used in this work is presented in the last part of the section.

### Dry Martini force field

Dry Martini is an implicit-solvent coarse-grained force field for lipids^31^ derived from the original explicit solvent Martini force field^23,80^. It is based on the same mapping with respect to an atomistic description of the lipids: approximately 4 atoms form a CG bead. The force field consists of bonding and non-bonding interaction terms where the former include only bond and angular potentials while the latter include van der Waals and electrostatic interactions. The van der Waals interactions are described by standard Lennard-Jones (LJ) potentials having the parameters re-calibrated with respect to the original Martini model in order to account for the lack of explicit solvation. Similarly some parameters of the bond and angular terms were re-parametrised to improve the properties of a simulated membrane bilayer. The electrostatic interactions are treated via a screened potential that is short-range in nature.

**Figure 10:**
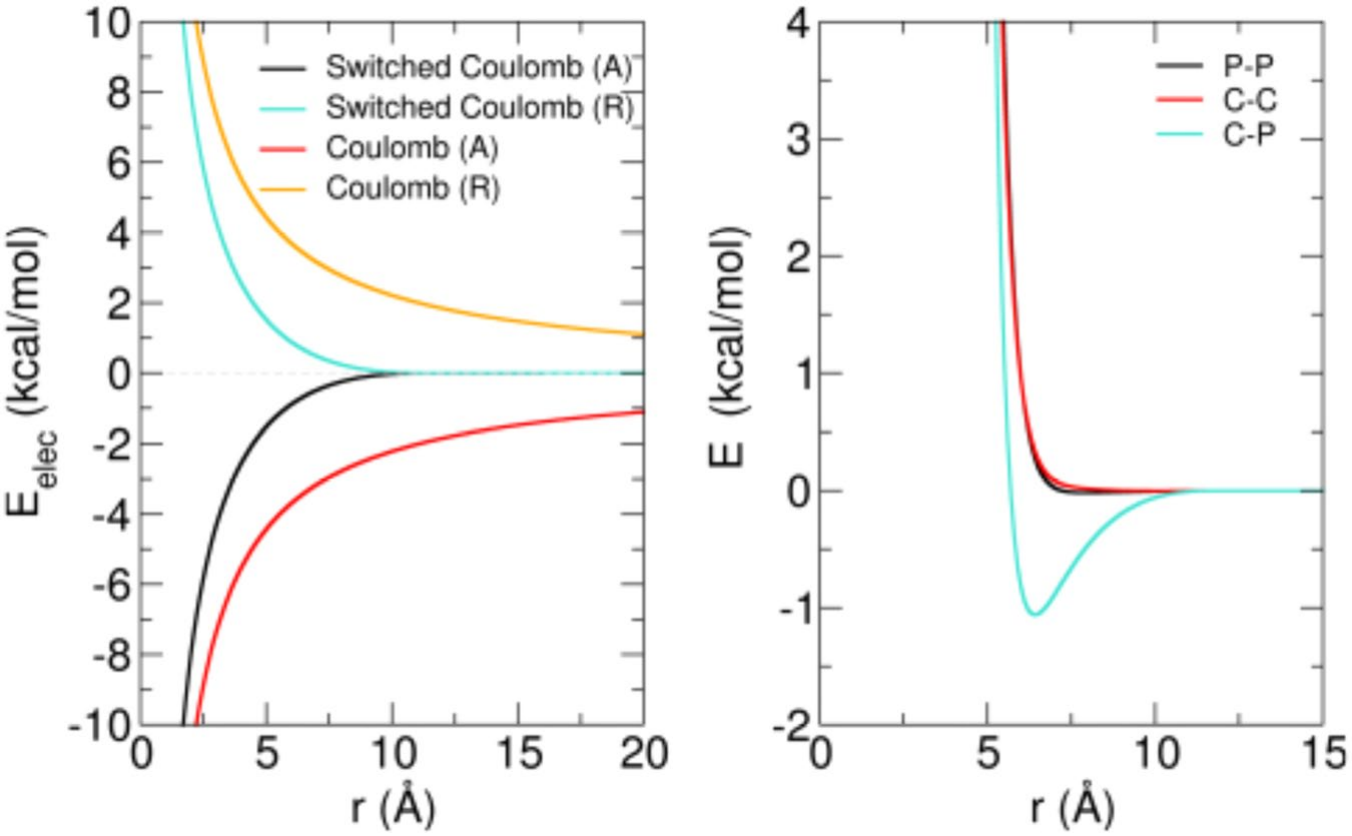
Non-bonded potentials. Left panel. Attractive (A) and repulsive (R) Coulomb potential with relative dielectric constant $\varepsilon =15$ compared to the switched electrostatic potentials used in the Dry Martini force field to describe the electrostatic interactions among charged groups. Right panel. Tabulated potentials that incorporate the electrostatic switched interactions and the short-range LJ interactions for the phosphate (P) and choline (C) charged groups of the lipids.

In order to properly describe the implementation of the Dry Martini model in our simulation package, MUPHY^53^, two features of the force field must be highlighted. First, concerning the angular term of the Hamiltonian, Dry Martini uses a very soft potential of the form ${U}_{\theta }=\frac{1}{2}{K}_{\theta }{\cos }^{2}(\theta -{\theta }_{0})$, which is different from standard harmonic ones. The second aspect concerns electrostatic interactions: instead of considering a standard long-range $1/r$ potential, the interactions between charged groups are described by a screened short-range potential. The screening is encoded in a polynomial switch function acting on the potential from $r=0$ up to the cut-off ${r}_{c}$ set equal to $12$ Å. The comparison between standard and switched Coulombic potentials is provided in the left panel of Fig. 10. The screening is intended to account for the lack of explicit solvation and ionic strength in the implicit solvent description^31^. In our implementation, we use tabulated potentials for the interactions between charged groups, adding together the screened electrostatic and the LJ terms (see right panel in Fig. 10).

### Lattice Boltzmann MD

In LBMD, the particle dynamics is coupled to the kinetic representation of the solvent, simulated via the Lattice Boltzmann (LB) technique^81^. This coupling allows to naturally include hydrodynamics in the simulation of biomolecules represented via implicit-solvent models. The coupling between particles and solvent arises from a Stokes-like drag force acting on each particle:

1 $${\overrightarrow{F}}_{i}^{D}=-\gamma ({\overrightarrow{v}}_{i}-{\overrightarrow{ {\tilde{u}} }}_{i})$$

where ${\overrightarrow{v}}_{i}$ is the $i$-th particle velocity, ${\overrightarrow{ {\tilde{u}} }}_{i}$ is the fluid velocity $\overrightarrow{u}$ smeared over a finite extension of the $i$-th particle, and $\gamma$ is the frictional coupling, an adjustable parameter in the methodology. The drag force adds up to the usual conservative forces derived from the Hamiltonian of the system, ${\overrightarrow{F}}_{i}^{C}={-\overrightarrow{\nabla }}_{i}U(\{r\})$ and to a random white noise, ${\overrightarrow{F}}_{i}^{R}$, that represents thermal fluctuations. The reader can find more technical details in refs^36,43^.

In our scheme, the LB implementation uses the BGK (Bhatnagar-Gross-Krook) collisional operator^40^, with a lattice grid spacing of $3$ Å, sufficient to resolve local hydrodynamic interactions for macromolecular systems. In our previous works^36,43^ we have provided a detailed discussion about the optimal size of the grid spacing to achieve an efficient coupling of LB with CG bio-molecule representations. The LB kinematic viscosity ${\nu }_{0}$ was set to reproduce bulk water behaviour at ambient conditions. In this work, the molecular and fluid dynamics were evolved synchronously using a physical timestep of $10$ fs, a value dictated by the stiffness of the molecular forces. In some simulations, we explored the effect of different values of the friction coefficient $\gamma$ in order for instance to tune protein diffusion in crowded conditions. Moreover when exploring the size effect on lipid diffusion in membranes we performed the simulations using a multiple-timestep scheme. The friction coefficient used for the lipids was $\gamma =0.00025$ fs^−1^ ^31^. In the present implementation all the simulations were performed using a constant volume/constant temperature scheme (NVT).

### Systems and simulation setups

All simulations were performed using the code MUPHY^53^. For verification purposes, comparisons with the original Dry Martini implementation in Gromacs^79,82^ were performed. The setup of the studied systems is provided below.

#### Bilayers

The POPC lipid bilayers were generated using the tool Insane provided with Martini^83^. A total of $1{0}^{5}$ steps of steepest descent minimisation followed by NVT equilibration lasting $50$ ns were performed with the Gromacs software (v 4.5.5). The equilibrated systems were used to start the LBMD simulations. In order to study system size dependence of the diffusivity of the lipid molecules in the membrane we have assembled several bilayers with lateral sizes ${L}_{x}={L}_{y}$ ranging from $100$ Å up to $400$ Å. All assembled systems have a surface area per lipid of $69.4$ Å${}^{2}$.

#### Nanotube

To simulate the adsorption of a carbon nanotube into a membrane we followed the approach designed by Bhaskara *et al*.^84^ based on the Martini force field. The tube had a diameter of $15$ Å and was assembled by stacking 11 “carbon” rings each constituted by 11 beads. The distal rings were formed by hydrophilic particles while the inner rings were formed by hydrophobic beads. The total length of the nanotube was $L=43$ Å.

#### Vesicles and Nanoreactors

The vesicles simulated in this work were built with the help of the Martini vesicle maker utility in the CHARMM-GUI webserver^85^ using the Dry Martini force field and equilibrated in Gromacs (v 5.1.4) following the CHARMM-GUI protocol. We built four systems of increasing size with initial vesicle radius equal to $r=50$ Å, $60$ Å, $100$ Å and $160$ Å, respectively (the radius is defined from the vesicle centre up to the centre of the lipid bilayer).

The smallest system and the vesicle of $R=100$ Å were used to investigate the dynamics of a vesicle under shear flow as a function of the shear rate. The vesicle of $R=60$ Å was filled by 15 small tri-peptide molecules (alanyl-phenylalanyl-alanine) in order to reproduce substrate evacuation under the action of shear flow. The substrate molecules were added inside the vesicle using the Packmol software^86^. The largest vesicle was filled with 5 $\alpha$-chymotrypsin enzymes, 6 crowding bovine serum albumin (BSA) proteins, and 100 substrate molecules (alanyl-phenylalanyl-alanine) in order to generate a model of a nanoreactor and to investigate protein-substrate encounters under the action of confinement, crowding and HI. The vesicle was filled using Packmol^86^. The structures of the enzyme ($\alpha$-chymotrypsin, PDB code 1YPH) and of the crowder proteins (BSA, PDB code 4F5S^87^) were obtained from the PDB database^88^. In the intermediate and largest systems, proteins and peptides were modelled using an elastic network (EN) based on C${}_{\alpha }$ and side-chain (SC) beads with a distance cutoff ${r}_{c}=8$ Å and a force constant $k=5$ kcal/(mol Å^2^). The inter-molecular interactions were based on the OPEP v.4 force field^89^. In the EN the size of C${}_{\alpha }$ beads was increased by 50% compared to the standard OPEP model. This choice was motivated by the fact that in the EN model the backbone of a residue is reduced to the C${}_{\alpha }$ bead only. In the case of small peptides like the substrate, because of their small size, the C${}_{\alpha }$ beads can partially penetrate inside the external fold of the crowders altering the diffusivity. Our rescaling prevents this incorrect behaviour. The OPEP SC-SC non-bonded interaction energies were rescaled by $f=0.545$, a factor which we found to describe more accurately the structure and dynamics of crowded protein solutions. The non-bonded interactions of the lipids with the content of the vesicle were parameterised by utilising existing OPEP potentials rescaled by the same factor $f$. More specifically, the positively charged choline group of POPC was treated as an OPEP side-chain bead of lysine for the interactions with amino acid beads, the negatively charged phosphate group as that of an OPEP glutamate bead, the two polar glycerol beads as the OPEP asparagine side-chain bead, and finally, the apolar beads of the POPC tails each as the OPEP leucine side-chain bead. In the simulation the friction coefficient was set to $\gamma =0.0018$ fs^−1^ for $\alpha$-chymotrypsin, $\gamma =0.001$ fs^−1^ for BSA, and $\gamma =0.05$ fs^−1^ for the substrate in order to reproduce the respective HYDROPRO^90^ diffusion coefficients in dilute conditions.

## Supplementary information

Supplementary Information
