# Supplementary material for: Modelling lipid systems in fluid with Lattice Boltzmann Molecular Dynamics simulations and hydrodynamics
Source: Sci Rep. 2019 Nov 11;9:16450. doi: 10.1038/s41598-019-52760-y (PMC6848203; doi:10.1038/s41598-019-52760-y)
Supplement: Supplementary file 1 — Supplementary Information [file 41598_2019_52760_MOESM1_ESM.pdf]

Supporting Information. Modelling lipid systems in fluid with Lattice Boltzmann Molecular Dynamics simulations and hydrodynamics

Astrid Brandner[1], Stepan Timr[1], Simone Melchionna[2,3], Philippe Derreumaux[1], Marc Baaden[1], and Fabio Sterpone[1,*]

[1]Laboratoire de Biochimie Théorique, IBPC, CNRS UPR9080, Univ. Paris Diderot, Sorbonne Paris Cité, 13 rue Pierre et Marie Curie, 75005, Paris, France
[*]fabio.sterpone@ibpc.fr
[2]ISC-CNR, Dipartimento di Fisica, Università Sapienza, P.le A. Moro 5, 00185, Rome, Italy
[3]Lexma Technology 1337 Massachusetts Avenue, Arlington, MA 02476, USA

ABSTRACT

Table S1. Nanotube adsorption times obtained in 17 different simulations with and without hydrodynamics.

System	τ [ns] (no HI)	τ [ns] (HI)
S1	8.20	6.37
S2	14.89	10.91
S3	7.09	6.41
S4	6.17	5.02
S5	12.34	12.36
S6	9.90	6.71
S7	10.62	10.95
S8	15.09	11.92
S9	7.14	9.87
S10	9.15	14.21
S11	37.81	37.97
S12	30.23	29.5
S13	21.33	30.77
S14	37.36	29.22
S15	34.95	20.9
S16	22.18	31.23
S17	28.79	10.52

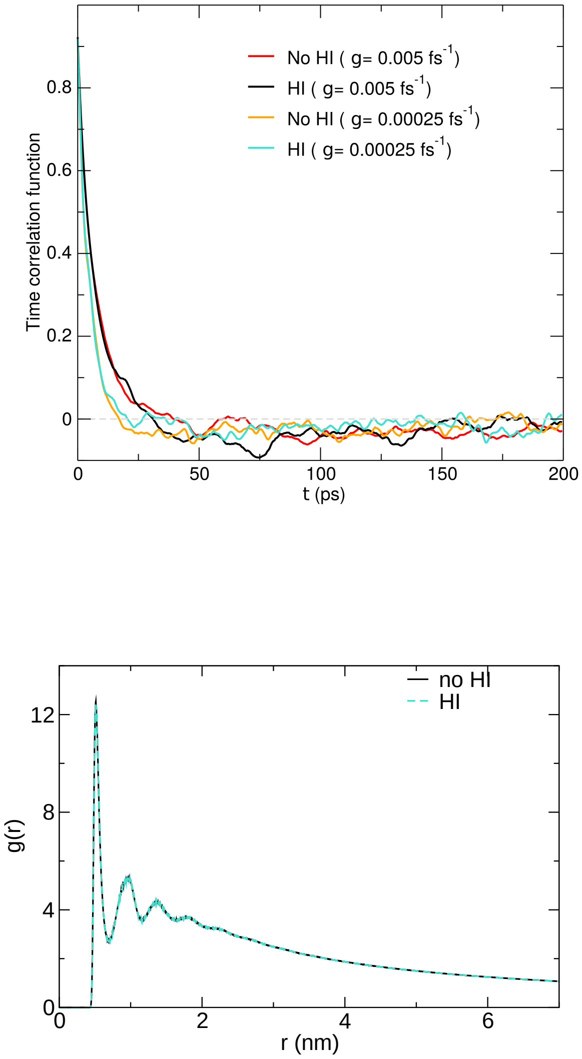

Figure S1. Top. Time correlation function of the membrane thickness fluctuation for a membrane system simulated with and without hydrodynamics, and using two different value of the friction coefficient γ. Bottom. Radial distribution function of hydrophobic beads for a simulation of a POPC bilayer with (HI, dotted cyan line) and without (no HI, black line) hydrodynamic interactions.

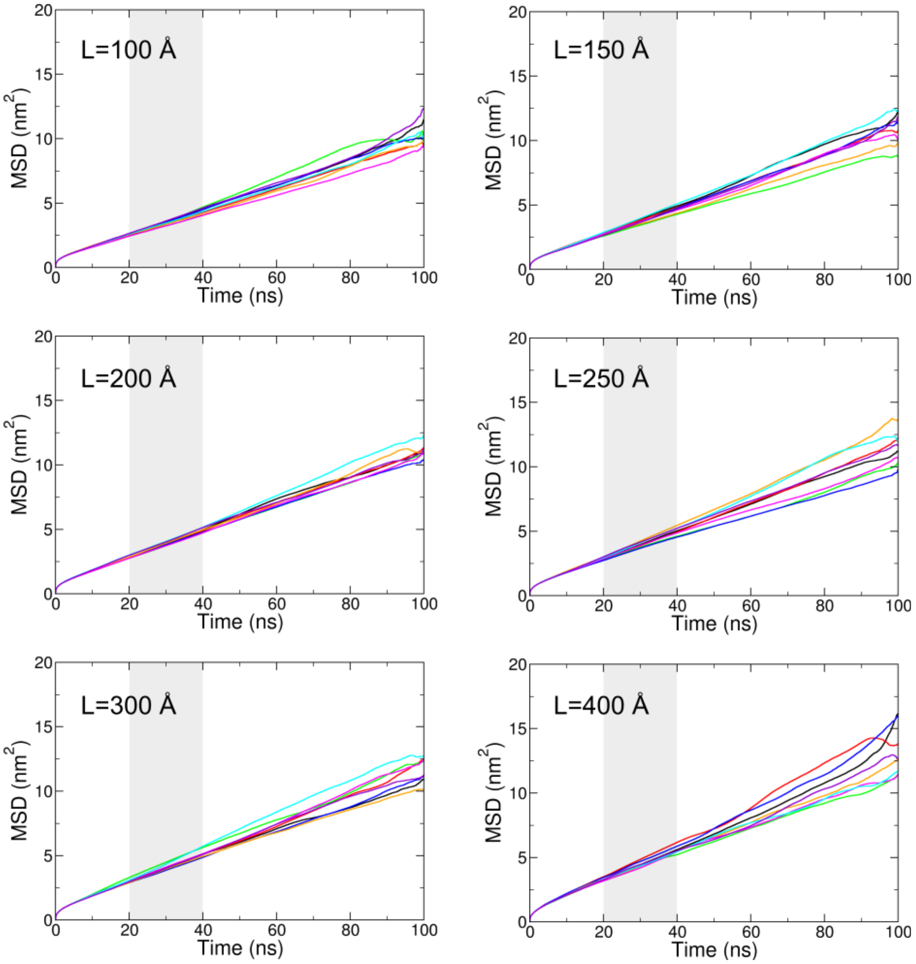

Figure S2. Lateral mean square displacement calculated (MSD) for the lipids in a planar membrane of size $L_x = L_y = L$ and fixed $L_z = 243$ Å. For each system the MSD is calculated using independent blocks of the trajectory, each of 100 ns. The diffusion coefficient was derived by a linear fit in the time window 20-40 ns (gray zone).

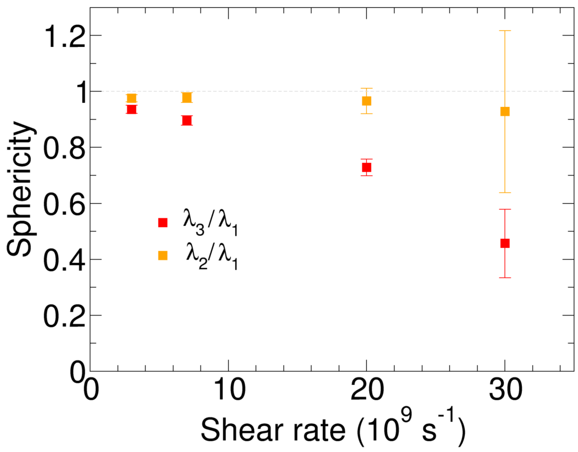

Figure S3. Vesicle sphericity computed as the ratio of the eigenvalue of the vesicle gyration tensor as a function of the shear rate.

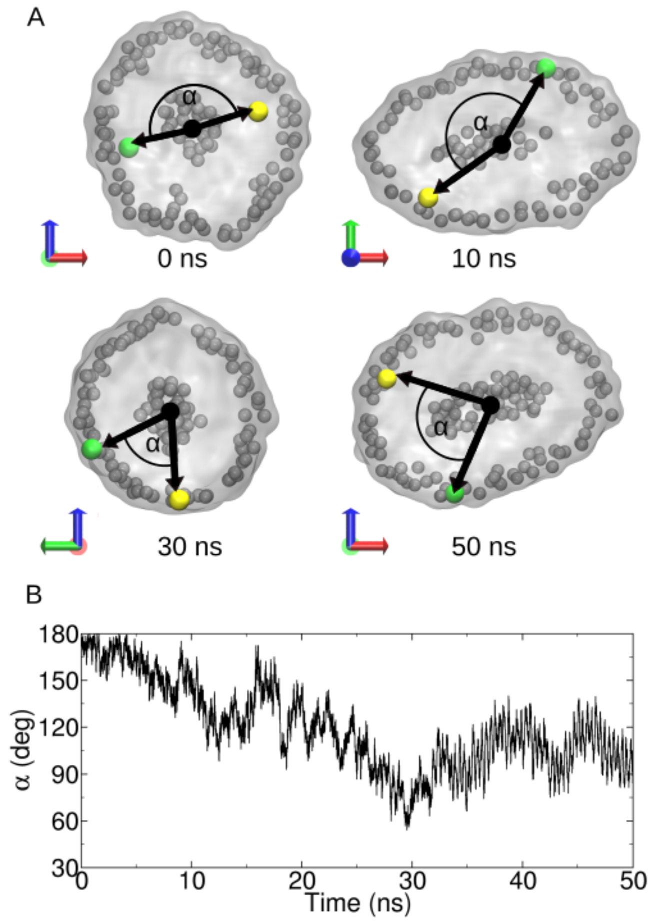

Figure S4. Time evolution of the angle formed between the polar heads of two lipids in the vesicle under the action of shear flow with $\dot{\gamma} = 20 \times 10^9 s^{-1}$.

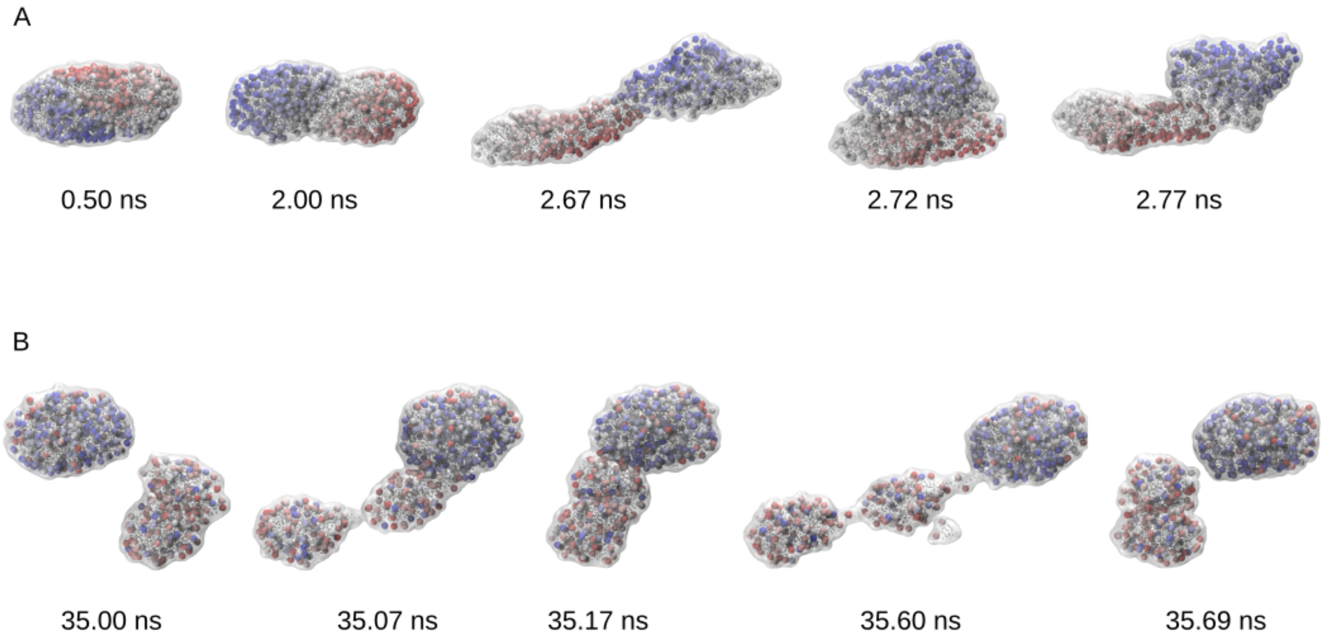

Figure S5. Vesicle under shear flow. At very high shear rate, $\dot{\gamma} = 30 \times 10^9 s^{-1}$, the vesicle breaks in smaller entities that encounter, fuse and disconnect as effect of the solvent shearing. The polar heads of two set of lipids, initially separated in the vesicle, were coloured in red and blue. The time evolution of the system shows their mixing.

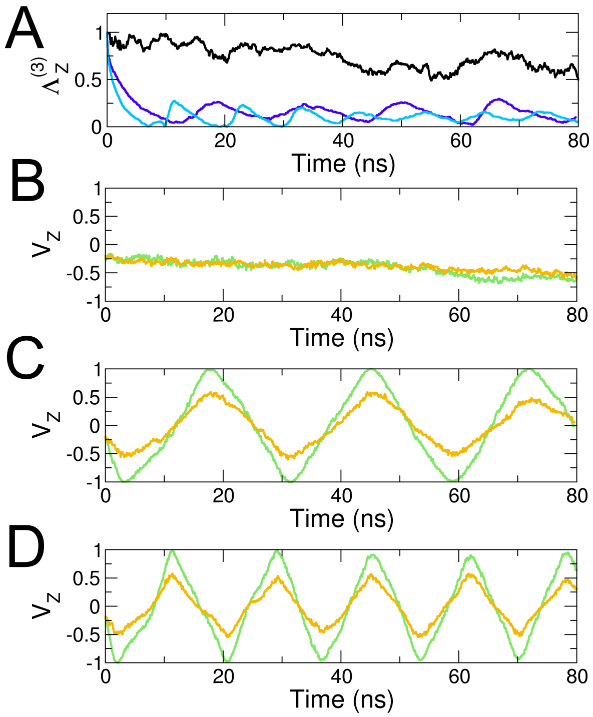

Figure S6. Vesicle under shear flow. In panel A we represent the time evolution of the normalised projection on the Z axis of the system of the eigenvector $\Lambda^{(3)}$ of the gyration tensor associated to the longest axis of the vesicle. The absolute value of the projection is reported in the figure for the three shear rates $\dot{\gamma} = 1.9$ (violet) and 3.7 (cyan) $\times 10^9 s^{-1}$ and $\dot{\gamma} = 3.7 \times 10^7 s^{-1}$ (black). Panel B-D: projection of the the vector **v** connecting one polar bead of the lipids in the membrane to the centre of mass of the vesicle. In each panel the green and yellow curves refer to two different beads in the membrane. Panel B refers to the lowest shear rate while Panel D to the highest.
